# Field performance of grafted, micropropagated, and own-rooted plants of three Italian hazelnut cultivars during the initial four seasons of development

**DOI:** 10.3389/fpls.2024.1412170

**Published:** 2024-06-12

**Authors:** Chiara Traini, Simona Lucia Facchin, Raffaella Brigante, Alessandra Vinci, Sofia Persichetti, Massimiliano Meneghini, Maurizio Micheli, Franco Famiani, Silvia Portarena, Giuliano Dradi, Daniela Farinelli

**Affiliations:** ^1^ Dipartimento di Scienze Agrarie, Alimentari e Ambientali, Università degli Studi di Perugia, Perugia, Italy; ^2^ Vivai Piante Battistini Società Agricola S.S, Cesena, FC, Italy; ^3^ Consiglio Nazionale delle Ricerche (CNR), Istituto di Ricerca sugli Ecosistemi Terrestri, (IRET), Porano, TR, Italy

**Keywords:** *Corylus avellana* L., Tonda Giffoni, Tonda Francescana®, Tonda Romana, growth rate, canopy volume, Relative growth Rate (RGR)

## Abstract

**Introduction:**

Over the course of four consecutive years, a comparative study, for the first time, was carried out to assess their growth characteristics, vegetative and productive performances.

**Material:**

Micropropagated, grafted on not suckering rootstock and own-rooted plants by layering from three Italian hazelnut (*Corylus avellana* L.) cultivars were established in the same orchard and environmental condition.

**Results:**

We found that the micropropagated plants, regardless of the variety considered, even being smaller than the other plants at the beginning of the plantation, reached similar sizes as the other plants after four growing seasons. Furthermore, micropropagated plants exhibited greater uniformity in growth compared to grafted ones, while own-rooted plants displayed more variability. No significant differences in yield performance and canopy volume were observed among the three propagation methods. These results suggest that the *in vitro* propagation technique, even in hazelnut, allows standardizing the plant material while preserving cultivar characteristics. Finally, *in vitro* propagation as well as grafting can be safely recommended for the cultivation of hazelnut cultivars.

## 1 Introduction

The European hazelnut (*Corylus avellana* L.) is an important species, a native shrub, widespread in the temperate zones of the northern hemisphere, which is rapidly spreading all over the world, especially in terms of production (t per year). Leading producers include Turkey (765,000 t), Italy (98,670 t), Azerbaijan (72,104 t), the USA (70,310 t), Chile (62,557 t), Georgia (33,400 t), and China (24,695 t) according to FAOSTAT data, 2022. Thanks to the high suckering capacity, the most common propagation method for hazelnut is layering, a technique that takes advantage of the plant’s ability to produce suckers from buds located at the base of the trunk (Malvicini et al., 2009). Also, the sucker emission can be considered as an opportunity to renovate the orchard, so it acquires an economic value in hazelnut orchard management (Pacchiarelli et al., 2022). In the Italian tradition, the propagation of hazelnut plants was always done, at least until a few years ago, using suckers taken from “vigorous mother plants” selected in hazelnuts cultivated to produce fruits (Bacchetta et al., 2008). Therefore, the oldest orchards have been set up using the material propagated from commercial orchards. This method is very easy to apply, but also, it is characterized by some negativities, such us the impossibility to guarantee a healthy propagation material, uncertainty of varietal identity, not being possible to distinguish seedlings from own-rooted suckers, and more suckering plants (Bacchetta et al., 2008). In order to verify the genetic origin and the phyto-pathological quality, starting from selected and certified mother plants, 15 years ago, following the nursery techniques observed and studied in the United States, the hazelnut propagation has been improved also in Northern Italy through the use of the stump layering, just to obtain rooted suckers (Roversi, 2015). However, this method requires a large number of mother plants, making it difficult to ensure that the propagation material is healthy. In addition, this method has many drawbacks both from the point of view of genetic matching (Bacchetta et al., 2008; Malvicini et al., 2009).

Over time, other propagation techniques have spread, such as grafting on no suckering rootstocks and, more recently, micropropagation (Avanzato et al., 2007; Prando Sandoval et al., 2014; Rovira, 2021). Grafting on no suckering rootstocks, such as open-pollinated or clonal *Corylus colurna* L., helps produce sucker-free plants, enhancing orchard management and mechanization (Rovira, 2021; Portarena et al., 2022). Additionally, grafted plants typically exhibit deeper root systems, enhancing drought/frost resistance, longevity, vigor, and productivity (Rovira, 2021; Portarena et al., 2022). Furthermore, the grafted trees are long-lived and adapted to a wide range of soil conditions. The *in vitro* propagation by tissue culture or micropropagation as a method of vegetative propagation of plant or fruit species offers many advantages such as fast propagation of uniform clones (Yu and Reed, 1995; Nas and Read, 2004; Leposavić et al., 2016). Moreover, micropropagation guarantees the production of healthy material of certain genetic origin and is a valid method for large-scale multiplication of certified material (Micheli et al., 2018; Bettoni et al., 2022; Leto et al., 2023; Bettoni et al., 2024) and of new cultivars obtained from breeding programs (e.g., Tonda Francescana®) (Bacchetta et al., 2008; Prando Sandoval et al., 2014; Silvestri et al., 2020). Therefore, this technique offers a viable alternative to traditional propagation methods, as it enables the production of a large number of plants in a confined space and independent of seasonal constraints (Micheli et al., 2019).

Field studies comparing micropropagated plants with other plants from different propagation systems, such as grafted ones, have already been carried out in species such as peach trees (Hammershing and Scorza, 1991), apple trees (Maguylo and Lauri, 2004), and walnut trees (Hasey et al., 2004).

In hazelnut cultivation, Valentini et al. (2009) assessed different training systems in own-rooted, but not micropropagated plants. In contrast, researchers in Spain and Italy have compared own-rooted plants with grafted ones (Rovira, 2021; Portarena et al., 2022; Rovira et al., 2022). To the best of our knowledge, no studies have yet compared micropropagated plants with grafted and own-rooted *Corylus avellana* L. plants. Studies in other plant species, such as olive, have found that micropropagated plants sometimes exhibit phenotypic differences when cultivated in the field due to epigenetic and genetic variations that occur during tissue culture processes (Leva et al., 2002). Data on these aspects are not yet available for hazelnut. Therefore, the aim of this study is to compare, for the first time, the vegetative growth and early productive performance of hazelnut plants obtained through *in vitro* propagation (later micropropagated plant), by English double cleft winter grafting on *Corylus colurna* L. rootstock (later grafted plant) vs. a traditional propagation system, stump layering (later own-rooted plant). The comparison was conducted in the same orchard and environmental conditions, from transplantation to the end of the fourth growing season.

## 2 Materials and methods

### 2.1 Environment, orchard characteristics, and sampling

This study was carried out in experimental hazelnut orchard located in Deruta (PG), in central Italy (42°97′26.00′′N, 12°40′32.4′′E) at 163 m a.s.l., managed by the University of Perugia. The experiment was conducted from February 2020 to the February 2024, from planting until the end of the fourth growing season.

Herein, the year 2020 is named as first growing season, the year 2021 as second growing season, the year 2022 as third growing season, and the year 2023 as fourth growing season.

**Figure 1:**
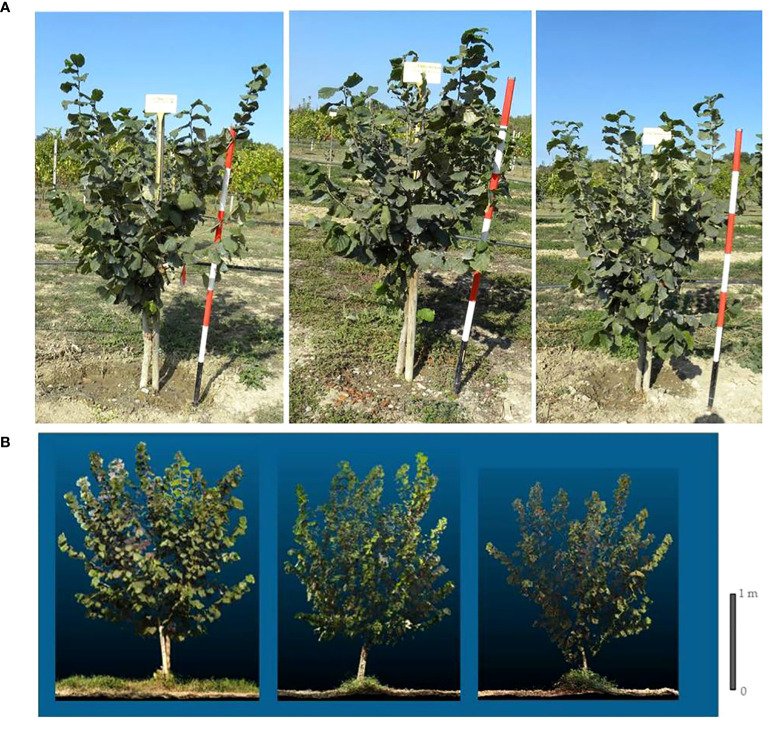
Grafted plant (left), micropropagated plant (centre) and own-rooted plant (right) of T. Francescana cv. at third (top) **(A)** and at fourth growing season (at the bottom) **(B)**.

**Figure 2:**
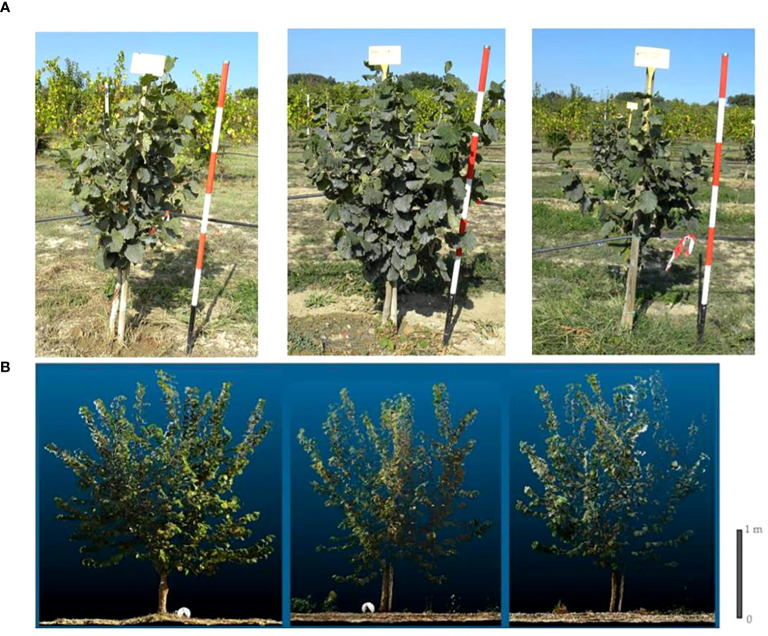
Grafted plant (left), micropropagated plant (centre) and own-rooted plant (right) of T Giffoni cv. at third (top) **(A)** and at fourth growing season (at the bottom) **(B)**.

**Figure 3:**
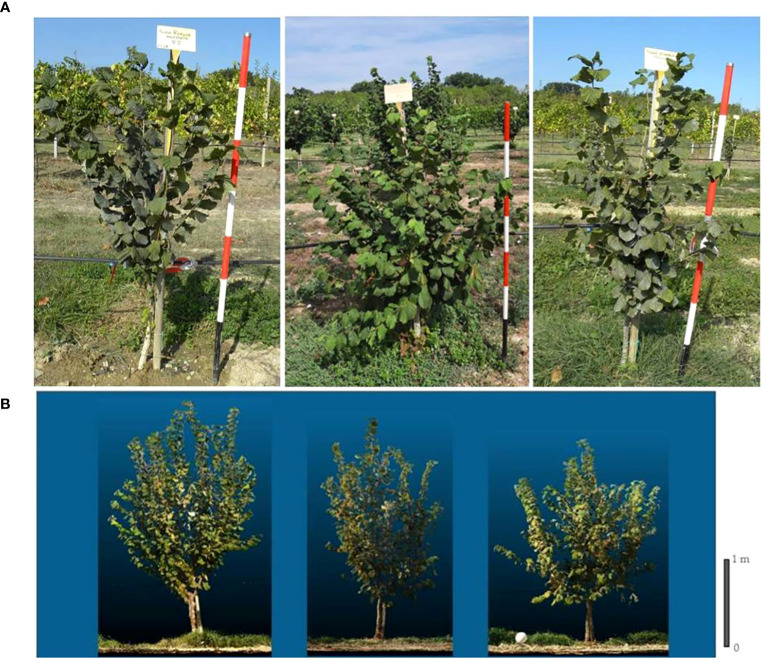
Grafted plant (left), micropropagated plant (centre) and own-rooted plant (right) of T. Romana cv. at third (top) **(A)** and at fourth growing season (at the bottom) **(B)**.

The orchard was implemented in February 2020 with 714 trees ha^−1^, spaced 4 m between rows and 3.5 m on the row, trained with a single trunk. The trees were planted applying a split plot experimental design, where the main treatment was represented by three types of plant material obtained through *in vitro* propagation, by English double cleft winter grafting on *Corylus colurna* L. rootstock and by stump layering. The second treatment was represented by three of the main Italian hazelnut cultivars, namely, Tonda di Giffoni (later T. Giffoni), Tonda Romana (later T. Romana), and the recently released Tonda Francescana® (later T. Francescana). For each type of plant material, 27 plants were used, with 9 plants used for each variety. Each plot consisted of nine plants of the same type of plant material, with three plants per cultivar. The orchard was irrigated and managed according to good agricultural practices. The fertilization was applied, with increasing doses as plants grow, considering that the soil is sandy–silty, with a good amount of potassium and an average amount of phosphorus according to the recommendation for the crop (Tombesi, 1991). The soil was not grassed throughout the first 2 years, and later, from the third year, it was kept grassed between the rows and hoed under the rows. The climatic characteristics of the area were recently described by Vinci et al. (2023a). The images of the three types of plant (grafted, micropropagated, and own-rooted) in the three different studied cultivars are shown in **Figures 1**–**3**, taken at the third year and fourth years of the growing season.

### 2.2 Plant characterization

At the planting time, in January, and at the end of the first growing season (September), all the plants have been characterized by measurement of plant height and trunk diameter 30 cm above the ground, beyond the point of grafting in the case of grafted plants. At the end of the first growing season, the dead plants have been counted to determine the plantation failures. The uniformity of the plants was evaluated dividing the plant height in five size classes (40 cm–60 cm, >60 cm–80 cm, >80 cm–100 cm, >100 cm–120 cm, >120 cm) and the trunk diameter in four size classes (5 mm–10 mm, 10 mm–15 mm, 15 mm–20 mm, >20 mm) and calculating the percentage per each class of the plant. The same procedure was applied at the end of the first growing season, when the uniformity of the trunk diameter was assessed dividing the measured values in four size classes (<40 mm, >40 mm–50 mm, >50 mm–60 mm, >60 mm) and calculating the percentage per each class of the plant.

From the second growing season (year 2021), after performing for the first time after plantation the training pruning and applying of a single trunk training system, the plant characteristics were evaluated measuring trunk section and canopy volume. The trunk section, assimilated to a circle shape, was calculated using the section diameter, then circle radius, value measured with a digital gauge; it was measured at the end of the growing season, in fall. The canopy volume was assimilated to a cylindrical shape, measuring the thickness, width, and height of canopy, using a meter (Vinci et al., 2022; Vinci et al., 2023b).

To evaluate vegetative growth, the relative growth rate (RGR) (Lü et al., 2016; Zhang et al., 2019) and growth rate (GR) were used (Baldicchi et al., 2015). These indexes are usually used to describe the evolution of masses, but we believe that they can be useful to evaluate the dimensional changes of organs or as in this case of perennial trees.

RGR of plant height (PH), only in the first growing season, and RGR of trunk section (TS), per each growing season, was calculated as following:


$$\text{RGR}=\left(\text{ln }\left(\text{T}{\text{S}}_{\text{t}1}\right)-\text{ln}\left(\text{T}{\text{S}}_{\text{t}0}\right)\right)/\left({\text{T}}_{1}-{\text{T}}_{0}\right)$$

as the difference in the natural logarithm of the PH or TS values at time T_1_ (September) and at time T_0_ (January) expressed in days. RGR of PH was expressed as cm per day and RGR of TS as cm^2^ per day.

Growth rate (GR), expressed in percentage, was calculated as the difference of the PH or TS values at time T_1_ and T_0_, relative to the growing season, respect to values at the time T_0_


$$\text{GR}=\left(\text{T}{\text{S}}_{\text{t}1}\right)-\left(\text{T}{\text{S}}_{\text{t}0}\right)/\left(\text{T}{\text{S}}_{\text{t}0}\right)\times 100$$

Pruning was performed manually at the end of each growing season during wintertime by trained personnel, using pruning shears and pruning loppers. The quantity of pruned wood, per each type of plant material and cultivar, was determined by using wheelbarrow equipped with a balance.

The leaf area index (LAI) was assessed at the fourth growing season (year 2023) when the leaf foliage was completely developed using manual methods, according to the procedure described by Farinelli et al. (2005).

Starting from the third growing season (year 2022), yield per plant was determined by collecting all nuts by hands and by harvesting machine (Facma Mek 1800), in the fourth growing season (year 2023). Moreover, considering the yield reached at the fourth growing season, the plants were classified in production and not in production when they get more than 0.25 kg of nut in shell per tree or less than 0.25 kg of nut in shell per tree, and the percentage of plant in production and not in production was calculated.

Finally, tree growth habit of the trees (erect ones, semi-erect, or spreading canopy) was determined using lidar technology at the end of the fourth growing season (Bioversity, FAO and CIHEAM, 2008). The laser scans (Faro, Lake Mary, Florida USA) were conducted utilizing the Faro Focus 3D laser scanner, configuring a scanning resolution of 7.67 mm at a range of 10 m; the individual scans were subsequently processed using the Faro Scene software for alignment and creation of a single 3D point cloud in which geometric features of plants can be extracted.

The meteorological data were collected using a Spectrum (Thayer Court, Aurora) WatchDog 2000 Series Weather Station located close to the orchard.

### 2.3 Statistical analysis and principal component analysis

The data were statistically analyzed with ANOVA, and means were compared using the Student–Newman–Keuls test. Considering that the differences among the data are mainly due to growing more than to environmental conditions, statistical analysis was performed per type of plant material, per cultivar, and interaction.

Principal component analysis (PCA) was performed using the agronomical parameters as input variables to explore the variability among samples and to detect the most discriminating variables. PCA summarizes the information contained in the data matrix in fewer independent PCs, obtained as linear combinations of the original variables, lying in the direction of maximum variance (Farinelli et al., 2021). The data were statistically evaluated using the statistical environment NALISI and R (R Core Team, 2018).

## 3 Results

**Figure 4:**
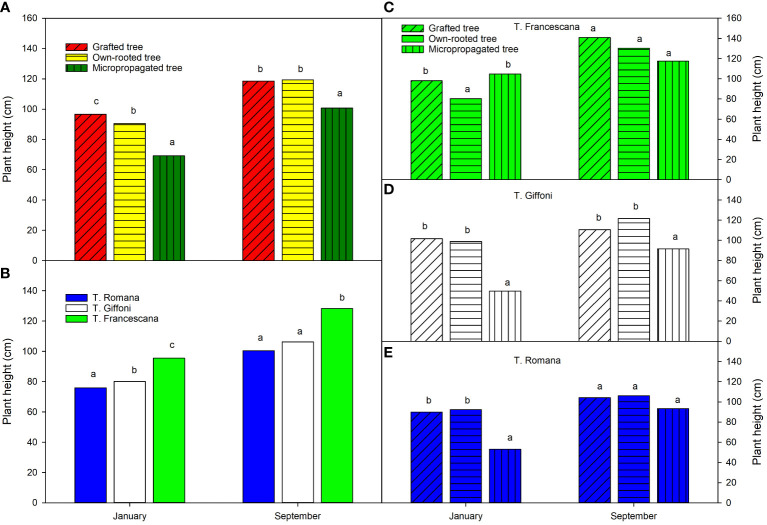
Plant height over the first growing season (year 2020) of the different type of plant material (left top) **(A)**, of the cultivars (left bottom) **(B)**, and interaction (right) **(C–E)**. Different lowercase letters indicate significant differences (P< 0.05), per each period, among the average values of type of plant and of cultivars.

At the time of transplanting (January), the grafted plants were the tallest (approximately 97 cm), followed by the own-rooted plants (approximately 90 cm), and the micropropagated plants were the smallest, with an average height of 69 cm (**Figure 4**, left top). The T. Francescana’s plants were the tallest (in average 96 cm), whereas the T. Romana’s ones the smallest (in average 76 cm) (**Figure 4**, left bottom). At the end of the first growing season (September), the grafted and own-rooted plants showed the same height (approximately 120 cm), whereas the micropropagated plants were still the smallest (approximately 100 cm) (**Figure 4**, left top). The plants of T. Francescana were still the tallest, and those of T. Romana had reached the same height of the T. Giffoni ones (**Figure 4**, left bottom). Analyzing how the plant height varied within each variety, it is observed that, at the beginning of the season, the smallest plants were the micropropagated ones, in the cultivars of T. Giffoni and T. Romana, respectively, and the own-rooted ones, in the cultivar T. Francescana (**Figure 4**, top right). At the end of the season, no statistically significant differences were observed in plant height among the propagation types for the T. Francescana and T. Romana cultivars. However, in the T. Giffoni cultivar, the micropropagated plants were still the shortest than the others (**Figure 4**, center right).

Regarding the uniformity of plant height, at transplant time, more than 90% of the grafted plants were higher than 80 cm (48.2% between 80 cm and 100 cm and 44.4% between 100 cm and 120 cm); around 81.5% of the own-rooted plants had a height of above 80 cm (66.7% between 80 cm and 100 cm and 14.8% between 100 cm and 120 cm) and 18.5% between 60 cm and 80 cm high. On the contrary, 70.8% of the micropropagated plants had a height between 40 cm and 60 cm and 25.9% >100 cm–120 cm (**Table 1**). In grafted plants, the most uniform plants were those of the cultivar T. Francescana, with 88.9% of plants between 100 cm and 120 cm height, followed by those of T. Romana with 77.8% of plants between 80 cm and 100 cm height (**Table 1**). In own-rooted plants, the most uniform plants were those of the cultivar T. Romana, with 88.9% of plants between 80 and cm 100 cm height, followed by those of T. Giffoni with 66.7% of plants between 80 cm and 100 cm height (**Table 1**). In micropropagated plants, T. Giffoni and T. Romana showed 100% of the plants between 40 and 60 cm height, whereas T. Francescana showed 77.8% between 100 cm and 120 cm height and 22.2% between 80 cm and 100 cm height (**Table 1**).

**Table 1:** Size classes (%/ tot) of plant height of grafted, micropropagated, and own-rooted plants of three Italian hazelnut cultivars at transplantation and at the end of the first growing season.

	At transplant (January)					At the end of the first growing season (September)				
Type of plant material /cultivar​	40 cm–60 cm​	60 cm–80 cm​	80 cm–100 cm​	100 cm–120 cm​	>120 cm​	40 cm–60 cm​	60 cm–80 cm​	80 cm–100 cm​	100 cm–120 cm​	>120 cm​
GRAFTED PLANT	0.0 B​	7.4​ A	48.2 B	44.4​ B	0.0​	0.0 A​	0.0 A​	14.8 A​	33.3 B​	51.9 B​
T. Giffoni​	0.0​ a	0.0​ a	55.6 b​	44.4 b​	0.0​	0.0​ a	0.0​ a	22.2 a​	33.3 a​	44.4 b​
T. Francescana​	0.0 a​	0.0​ a	11.1 a​	88.9 c​	0.0​	0.0​ a	0.0​ a	0.0 a​	0.0 a​	100 c​
T. Romana​	0.0​ a	22.2 b ​	77.8 b​	0.0 a	0.0​	0.0​ a	0.0​ a	22.2 a​	66.7 b​	11.1 a​
OWN-ROOTED​ PLANT	0.0 B​	18.5​ B	66.7 C	14.8​ A	0.0​	0.0 A​	0.0 A​	3.7 A​	44.4 B	51.9 B​
T. Giffoni​	0.0​ a	0.0 a	66.7​ a	33.3​ a	0.0​	0.0​ a	0.0​ a	0.0 a​	22.2​ a	77.8 b
T. Francescana​	0.0​ a	55.6 b​	44.4​ a	0.0​ a	0.0​	0.0​ a	0.0 a​	0.0 a​	33.3​ a	66.7 b​
T. Romana​	0.0​ a	0.0 a​	88.9​ a	11.1​ a	0.0​	0.0​ a	0.0​ a	11.1 a​	77.8​ b	11.1 a​
MICROPROPAGATED PLANT​	70.8 A​	0.0 A​	7.4 A​	25.9 A	0.0​	5.6 B​	25.9 B​	35.2 B​	1.9 A​	31.5 A​
T. Giffoni​	100.0 b​	0.0 a	0.0 a​	0.0 a​	0.0​	0.0​ a	11.1 a​	88.9 b​	0.0​ a	0.0 a​
T. Francescana​	0.0 a​	0.0 a​	22.2 b​	77.8 b​	0.0​	0.0​ a	0.0 a​	0.0 a​	5.6​ a	94.4 b​
T. Romana​	100.0 b​	0.0 a​	0.0 a​	0.0 a​	0.0​	16.7​ b	66.7 b​	16.7 a ​	0.0​ a	0.0 a

In each column, in type of plant and cultivar, means followed by different letters are significantly different per P< 0.05. Different lowercase letters indicate significant (P< 0.05) differences among the average values of cultivar. Different uppercase letters indicate significant (P< 0.05) differences among the average values of type of plant.

At the end of the first growing season, grafted and own-rooted plants showed a similar distribution with 33.3% and 44.4% of plant height between 100 cm and 120 cm and 51.9% over 120 cm height, respectively, whereas the micropropagated plants showed a larger distribution (**Table 1**). In fact, in micropropagated plants, 88.9% of the plants of T. Giffoni had reached a height between 80 cm and 100 cm, whereas 66.7% of the plants of T. Romana had a height between 60 cm and 80 cm and 94.4% of the plants of T. Francescana were over 120 cm in height (**Table 1**).

The grafted plants of the T. Giffoni cultivar showed a rather wide distribution from 80 to more than 120 cm, whereas all the plants of the T. Francescana cultivar were more than 120 cm tall, and 66.7% of those of the T. Romana cultivar were between 80 cm and 120 cm height (**Table 1**). 22% of T. Romana and T. Giffoni had a height between 80 cm and 100 cm. On the other hand, own-rooted plants of T. Giffoni and T. Francescana cultivar were fairly uniform in height with 77.8% and 66.7%, respectively, of plants over 120 cm height and 22.2% and 33.3% between 100 cm and 120 cm. The own-rooted plants of T. Romana were mainly tall between 100 cm and 120 cm (**Table 1**).

**Figure 5:**
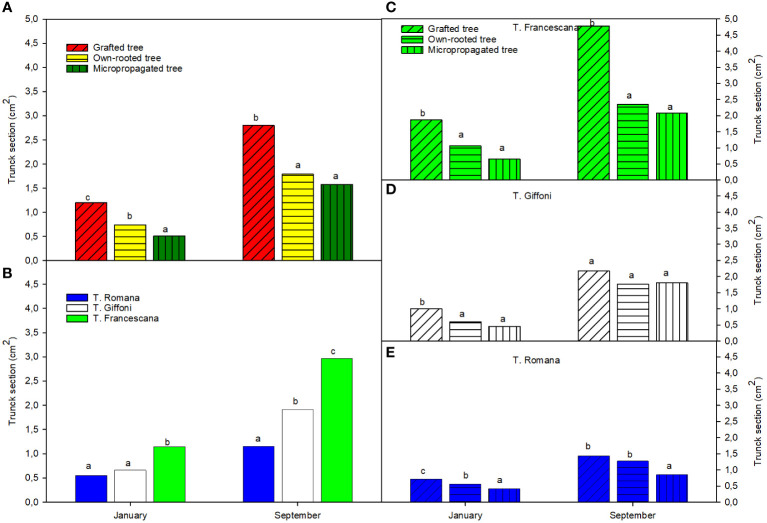
Trunk section over the first growing season of the different type of plant material (left top) **(A)**, of the cultivars (left bottom) **(B)**, and interaction (right) **(C–E)**. Different lowercase letters indicate significant differences (P< 0.05), per each period, among the average values of type of plant and of cultivars.

The micropropagated plants were the smallest in terms of trunk section (around 0.5 cm^2^) compared with the own-rooted ones (around 0.6 cm^2^) and grafted ones (approximately 1.2 cm^2^) (**Figure 5A**, left top). Among the cultivar, T. Francescana showed the largest trunk section (approximately 1.0 cm^2^) than the T. Giffoni (around 0.6 cm^2^) and the T. Romana (around 0.5 cm^2^) (**Figure 5**, left bottom). Therefore, among the varieties, the trend observed about plant height has been confirmed also for the trunk section, meaning that F. Francescana showed the highest values, followed by T. Giffoni, whereas plants of T. Romana resulted to be always the smallest.

On the contrary, at the end of the growing season, micropropagated plants reached the same trunk section dimensions of the own-rooted ones (around 1.5 cm^2^), but they were found to be still smaller than the grafted plants (around 3.0 cm^2^) (**Figure 5**, left top). Among the varieties, regardless of type of plant material, the T. Francescana was confirmed to be the one with the largest trunk section size (approximately 3.0 cm^2^) (**Figure 5**, left bottom). Evaluating the different types of plant material in each cultivar, in the T. Giffoni, no statistically significant differences have emerged, whereas in T. Francescana, the grafted plants were found to be greater (approximately 5 cm^2^) than the other two types (respectively 2.5 cm^2^ for the own-rooted and 2.0 cm^2^ for the micropropagated ones) (**Figure 5**, center and top right). Moreover, only in the case of the variety T. Romana, the micropropagated plants have shown to have smaller dimensions than both other plant types (**Figure 5**, right bottom).

Furthermore, evaluating the uniformity of trunk diameter (**Table 2**), at the time of transplantation, in January, 96.3% of micropropagated plants and 77.8% of own-rooted ones showed a trunk diameter between 5 mm–10 mm, whereas the grafted plants had a larger trunk size distribution (**Table 2**). At the end of the first vegetative season, regardless of the cultivars, the micropropagated plants have reached the same trunk size as the other two plant types, with only 13% of plant showing a trunk diameter smaller than 15 mm. Therefore, at the end of the first growing season, micropropagated plants, although they were smaller both in height and trunk section and diameter than those of the other two types of plant material, have almost filled the initial differences, showing significantly higher RGR and GR (**Table 3**). In specific, plant height RGR and GR of micropropagated plants were, respectively, 0.0016 cm day^−1^ and 57.7%, whereas those grafted and own-rooted showed 0.0008 cm day^−1^ and 0.0011 cm day^−1^ as RGR and 23.5% and 34.9% as GR (**Table 3**). The same trend and differences were observed regarding the RGR and GR of the trunk section (**Table 3**). As regards the cultivar, in grafted plants, T. Francescana showed the significant highest RGR and GR of plant height, as well as in own-rooted plants, whereas in micropropagated plants, the significant higher RGR and GR were observed in T. Giffoni and T. Romana cultivars (**Table 3**). The RGR and GR of the trunk section were similar in grafted plants of the three cultivars, significantly higher in own-rooted plants and micropropagated plants of T. Giffoni and T. Francescana cultivars, whereas those of T. Romana showed a slower RGR and GR (**Table 3**).

**Table 2:** Size classes (%/ tot) of trunk diameter of grafted, micropropagated, and own-rooted plants of three Italian hazelnut cultivars at transplantation and at the end of the first growing season.

	At the transplant (January)				At the end of first growing season (September)			
Type of plant material/cultivar​	5 mm–10 mm​	10 mm–15 mm​	15 mm–20 mm​	>20 mm​	5 mm–10 mm​	10 mm–15 mm​	15 mm–20 mm​	>20 mm​
GRAFTED PLANT	37,0 A	37,0 C	18,5 B	7,4 B	3.7 A	14.8 A	48.2 A	33.3 C
T. Giffoni​	33,3 b	66,7 b	0,0 a	0,0 a	0.0 a	11.1 ab	88.9 b	0.0 a
T. Francescana​	0,0 a	22,2 a	55,6 b	22,2 b	0.0 a	0.0 a	0.0 a	100.0 b
T. Romana​	77,8 c	22,2 a	0,0 a	0,0 a	11.1 a	33.3 b	55.6 b	0.0 a
OWN-ROOTED​ PLANT	77,8 B	18,5 B	3,7 A	0,0 A	0.0 A	55.6 C	37.0 A	7.4 B
T. Giffoni​	88,9 b	11,1 a	0,0 a	0,0 a	0.0 a	44.4 a	55.6 b	0.0 a
T. Francescana​	44,4 a	44,4 b	11,1 a	0,0 a	0.0 a	22.2 a	55.6 b	22.2 b
T. Romana​	100,0 b	0,0 a	0,0 a	0,0 a	0.0 a	100.0 a	0 a	0.0 a
MICROPROPAGATED PLANT​	96,3 C	3,7 A	0,0 A	0,0 A	13.0 B	35.2 B	50.0 A	1.9 A
Tonda Giffoni​	100,0 a	0,0 a	0,0 a	0,0 a	0.0 a	22.2 a	77.8 b	0.0 a
Tonda Francescana​	88,9 a	11,1 a	0,0 a	0,0 a	0.0 a	22.2 a	72.2 b	5.6 a
Tonda Romana​	100,0 a	0,0 a	0,0 a	0,0 a	38.9 b	61.1 b	0.0 a	0.0 a

In each column, in type of plant and cultivar, means followed by different letters are significantly different per P< 0.05. Different lowercase letters indicate significant (P< 0.05) differences among the average values of cultivar. Different uppercase letters indicate significant (P< 0.05) differences among the average values of type of plant.

**Table 3:** RGR and GR of plant height and trunk section of grafted, micropropagated, and own-rooted plants of three Italian hazelnut cultivars over the first growing season.

Type of plant material/cultivar​	RGR (cm day^−1^) Plant height	GR (%) Plant height ​	RGR (cm^2^ day^−1^) Trunk section	GR (%) Trunk section
GRAFTED PLANT	0.0008 A	23.5 A	0.0033 A	165.2 A
T. Giffoni​	0.0003 a	9.4 a	0.0032 a	145.5 a
T. Francescana​	0.0014 b	45.0 b	0.0041 a	242.6 a
T. Romana​	0.0006 a	16.1 a	0.0025 a	107.5 a
OWN-ROOTED​ PLANT	0.0011 A	34.9 A	0.0035 A	151.1 A
T. Giffoni​	0.0008 a	23.0 a	0.0042 b	199.0 b
T. Francescana​	0.0019 b	66.1 b	0.0031 a	123.0 a
T. Romana​	0.0005 a	15.8 a	0.0031 a	131.1 a
MICROPROPAGATED PLANT​	0.0016 B	57.7 B	0.0042 B	223.8 B
T. Giffoni​	0.0023 b	85.0 b	0.0055 b	328.5 b
T. Francescana​	0.0004 a	12.0 a	0.0045 b	234.2 b
T. Romana​	0.0021 b	76.1 b	0.0026 a	108.7 a

In type of plant and cultivar, means followed by different letters are significantly different per P< 0.05. Different lowercase letters indicate significant (P< 0.05) differences among the average values of cultivar. Different uppercase letters indicate significant (P< 0.05) differences among the average values of type of plant.

At the end of the first growing season, significant differences were observed among the plant types in terms of plant mortality; in fact, 8.3% of own-rooted plants were dead, whereas none of the other two plant types were dead. Regarding plant mortality within each cultivar studied, significant differences were found in T. Romana, where approximately 11% of own-rooted plants were dead, whereas no failure was noted in grafted and micropropagated plants.

**Figure 6:**
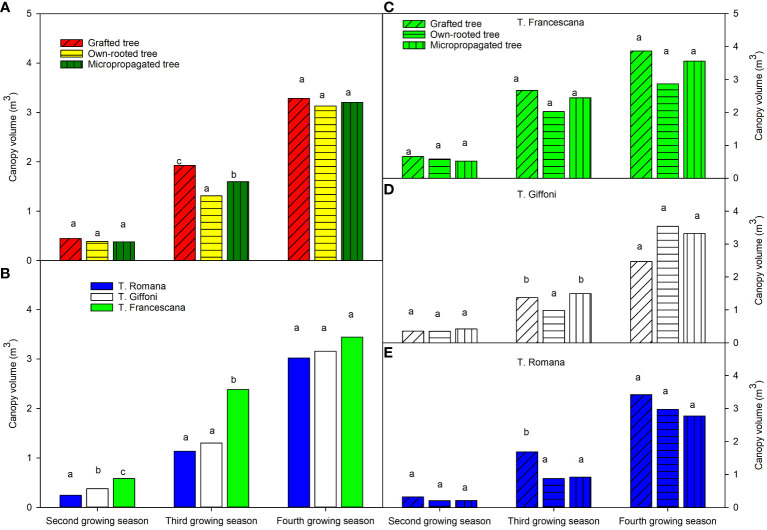
Canopy volume at the end of the second, third and fourth growing season of the different type of plant material (left top) **(A)**, of the cultivars (left bottom) **(B)**, and interaction (right) **(C–E)**. Different lowercase letters indicate significant differences (P< 0.05), per each growing season, among the average values of type of plant and of cultivars.

The canopy volumes of the plants, over the second growing season, were similar in the three types of plant material around 0.40 m^3^ (**Figure 6**, left top), whereas significant differences were among the three cultivars, showing T. Romana plants the smaller canopy volume and T. Francescana the bigger one (**Figure 6**, left bottom). At the third growing season, the canopy volumes were significant different among the three types of plant. In fact, the grafted plant showed the bigger canopy volume, followed by micropropagated one, whereas the own-rooted plants were the smaller, respectively, with values of 1.93 m^3^, 1.60 m^3^, and 1.31 m^3^ (**Figure 6**, left top). Over this time, the plants of T. Romana have reached the same canopy volume of those of T. Giffoni, whereas those of T. Francescana are still the biggest, around 3.2 m^3^ (**Figure 6**, left bottom). At the fourth growing season, the grafted plants showed the biggest canopy volumes, whereas the micropropagated and own-rooted plants had similar canopy volumes (**Figure 6**, left top).

Analyzing the development of canopy volume in each cultivar, from the second to the fourth growing season, it is observed that in the cultivar, T. Francescana canopy volume was similar in the three types of plants (**Figures 1**, **6**, right top). In the cultivar T. Giffoni instead, during the third growing season, the micropropagated plants and then also those grafted showed a more developed canopy than the own-rooted ones, but by the fourth year of growth, the three types of plants had a similar canopy (**Figures 2**, **6**, right center). In the cultivar T. Romana, in the third growing season, the grafted plants showed a more developed canopy than those own-rooted and micropropagated ones, and then in the fourth year, the plants had similar canopies in development (**Figures 3**, **6**, right bottom).

**Figure 7:**
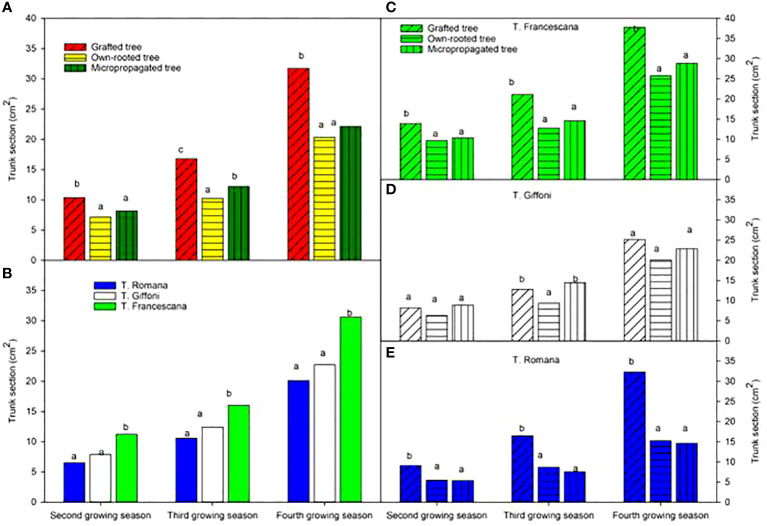
Trunk section at the end of the second, third and fourth growing season of the different type of plant material (left top) **(A)**, of the cultivars (left bottom) **(B)**, and interaction (right) **(C–E)**. Different lowercase letters indicate significant differences (P< 0.05), per each growing season, among the average values of type of plant and of cultivars.

The analysis also showed that, considering the cultivars, over the second and third growing seasons, T. Francescana cultivar showed greater growth than the other two cultivars (**Figure 1A**). On the contrary, the T. Romana cultivar resulted to be the one with the least growth at least up to the third growing season (**Figure 3A**). Regarding the trunk section, the grafted plants always showed a significantly bigger trunk section, varying from 10.4 cm^2^ at the end of the second growing season up to 31.7 cm^2^ at the end of the fourth growing season; on the contrary, those of the own-rooted and of micropropagated plants were approximately 7 cm^2^ and 20 cm^2^, respectively, and 8 cm^2^ up to 22 cm^2^, respectively (**Figure 7**, left top). The grafted plants in the T. Francescana and T. Romana cultivars had a larger trunk section than those other two plant types, whereas the grafted plants of T. Giffoni cultivar achieved a bigger trunk section only at the end of the second growing season (**Figure 7**, right center). Finally, T. Francescana had always a trunk section bigger than those of the other cultivars (**Figure 7**, left bottom).

**Figure 8:**
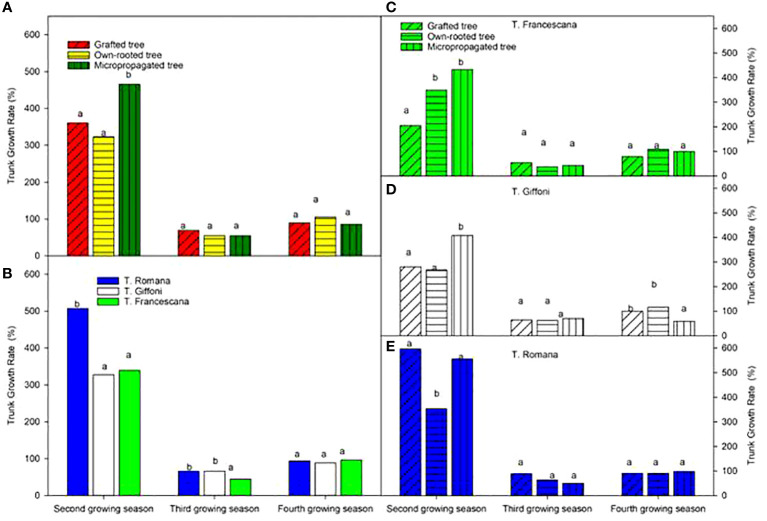
Growth rate of trunk section from the second to the fourth growing seasons of the different type of plant maternal (left top) **(A)**, of the cultivars (left bottom) **(B)**, and interaction (right) **(C–E)**. Different lowercase letters indicate significant differences (P< 0.05), per each growing season, among the average values of type of plant and of cultivars.

The micropropagated plants showed the highest growth rate of trunk section over the second growing season with respect to the grafted and own-rooted plants, whereas the three types of plant all had the same growth rate of the trunk (**Figure 8**, left top). These trends were confirmed for T. Francescana and T. Giffoni cultivars, whereas for T. Romana, the grafted plants showed not different GR than the micropropagated ones (**Figure 8**, right). T. Romana had a higher growth rate over the second growing season with respect the other two cultivars (**Figure 8**, right bottom).

**Figure 9:**
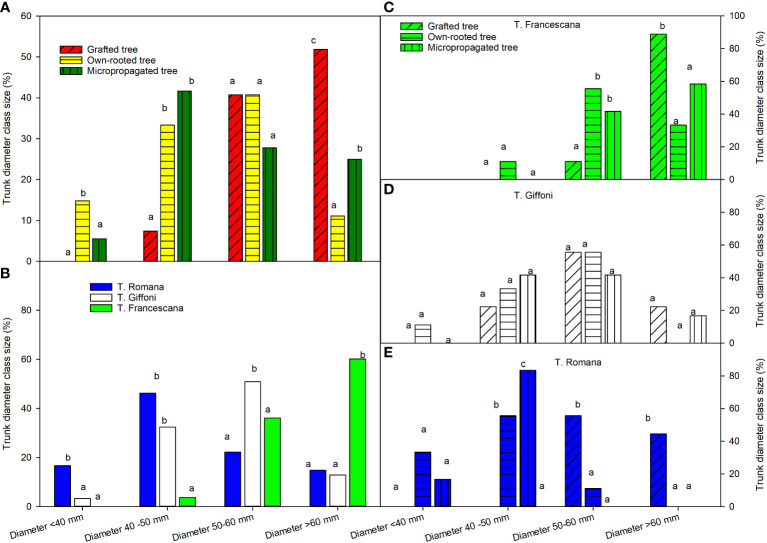
Trunk diameter class sizes at the end of fourth growing seasons of the different type of plant material (left top) **(A)**, of the cultivars (left bottom) **(B)**, and interaction (right) **(C–E)**. Different lowercase letters indicate significant differences (P< 0.05), per each class size, among the average values of type of plant and of cultivars.

At the end of the fourth growing season, grafted plants showed a trunk size much more uniform, than the other two plant types; in fact, their trunk diameters were only between 50 mm–60 mm and >60 mm, respectively, 41% and 52% (**Figure 9**, left top). Instead, the micropropagated plants were distributed among three class sizes and own-rooted plants among the fourth class size (**Figure 9**, left top). In the class 50 mm–60 mm, there were no differences among the types of plant material (**Figure 9**, left top).

Among the three cultivars, T. Francescana showed the highest trunk diameter uniformity, having over 95% of the plants with a trunk diameter over 50 mm, followed by T. Giffoni with only 63.9%; while T. Romana had a larger trunk size distribution, the majority of the plants had a trunk diameter in the class 40 mm–50 mm (46.3%) (**Figure 9**, left bottom).

Inside each cultivar, the plants of T. Giffoni were those with the greatest variability of the trunk diameter, whereas those of T. Francescana had less variability (**Figure 9**, right center and right top). Inside T. Romana cultivar, the grafted plants showed the highest values of plants with a trunk diameter over 50 mm, 55.6% between 50 mm and 60 mm, and 44% over 60 mm, whereas 83% of micropropagated plants showed a trunk diameter between 40 mm and 50 mm. The own-rooted plants showed 55.6% of trunk diameter between 40 mm and 50 mm; 33% less than 40 mm and 11% between 50 mm and 60 mm (**Figure 9**, right bottom).

**Figure 10:**
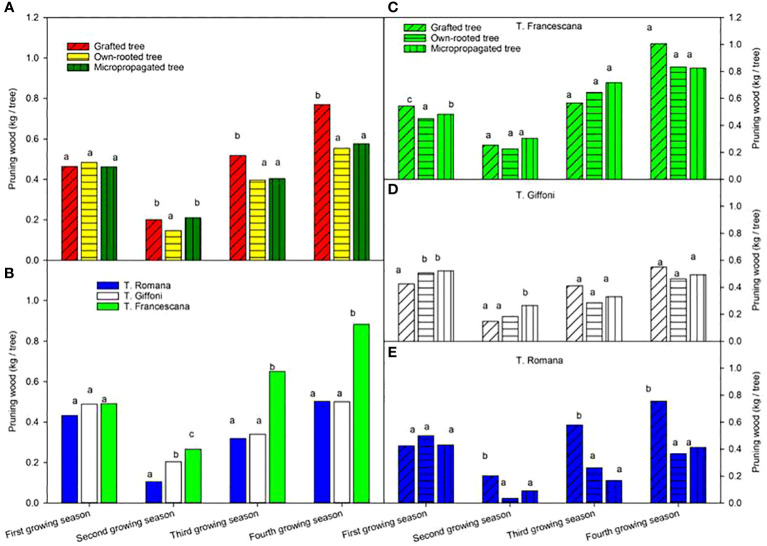
Pruning wood from the first to the fourth growing seasons of the different type of plant material (left top) **(A)**, of the cultivars (left bottom) **(B)**, and interaction (right) **(C–E)**. Different lowercase letters indicate significant differences (P< 0.05), per each growing season, among the average values of type of plant and of cultivars.

The pruning wood at the end of the first growing season varied from 0.43 kg to 0.49 kg per tree without any difference among plant types and among cultivars (**Figure 10**, left top and **Figure 10**, left bottom). At the end of the second growing season, the wood removed was less than those of the previous season, around 0.20 kg per tree, with small differences among plant types. At the end of the third and fourth growing seasons, the highest and significant quantity of pruned wood was removed from grafted tree, 0.52 kg and 0.77 kg per tree, respectively, whereas from own-rooted tree and micropropagated tree, it was, on average, around 0.40 kg and 0.57 kg per tree without significant differences (**Figure 10**, left top). As for cultivars, during the first season, the amount of wood removed by pruning was no different, whereas in the second year, the amount was significantly higher in T. Francescana, followed by T. Giffoni and T. Romana (**Figure 10**, left bottom). In the third and fourth growing seasons, the amount of wood removed was significantly greater in T. Francescana than the other two cultivars. Cultivar per cultivar, no significant differences were observed among the three types of plant of T. Francescana, except for the first year, where the wood removed from the grafted plants was significantly greater than that removed from the micropropagated plants and own-rooted plants (**Figure 10**, right top). For the T. Giffoni cultivar, at the end of the first growing season, wood pruned from micropropagated and own-rooted plants was higher than that from grafted ones, whereas at the end of the second growing season, wood pruned from micropropagated plants was significantly higher than those from the other two, 0.26 kg per tree and, on average, 0.17 kg per tree, respectively. The wood removed by pruning the following two seasons was not different as the quantity among the different plant types, on average 0.34 kg per tree and 0.54 kg per tree (**Figure 10**, right center). The wood pruned from grafted plant of T. Romana was always significantly higher than those removed from micropropagated and own-rooted ones, except for the first season (**Figure 10**, right bottom).

**Figure 11:**
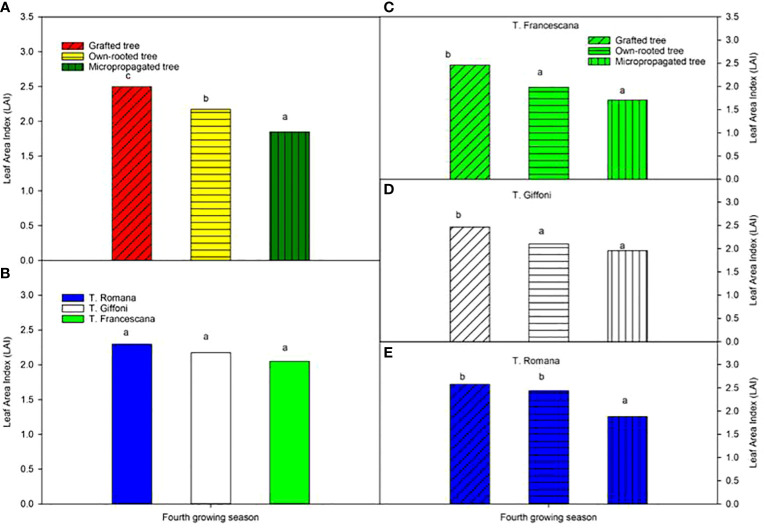
Leaf Area Index (LAI) at fourth growing seasons of the different type of plant material (left top) **(A)**, of the cultivars (left bottom) **(B)**, and interaction (nght) **(C–E)**. Different lowercase letters indicate significant differences (P< 0.05) among the average values of type of plant and of cultivars.

The leaf area index varied from 2.5, in grafted plants, to 1.9 in micropropagated plants, and it was different among the three types of plant material (**Figure 11**, left top), whereas LAI was similar among the three cultivars (around 2.2) (**Figure 11**, left bottom). Inside each cultivar, the grafted plants showed usually the significantly highest LAI value with respect to the other two types of plant, except in T. Romana where grafted plants had an LAI like that of own-rooted plants and significantly greater than that of micropropagated plants (**Figure 11**, right bottom).

At the fourth growing season, the grafted plants, regardless of the cultivars, showed an erect growth habit whereas the own-rooted and micropropagated ones resulted to have a semi-erect canopy (**Figures 1B**, **2B**, **3B**).

**Figure 12:**
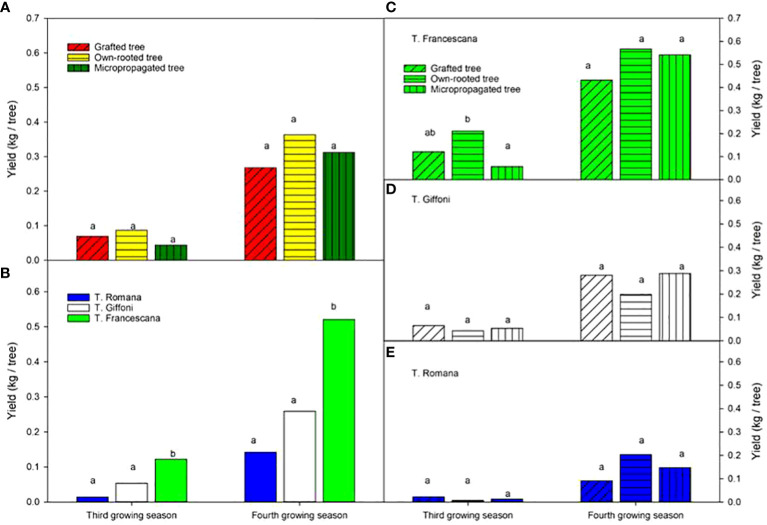
Yield recorded at the third and fourth growing seasons of the different type of plant material (left top) **(A)**, of the cultivars (left bottom) **(B)**, and interaction (right) **(C–E)**. Different lowercase letters indicate significant differences (P< 0.05), per each growing season, among the average values of type of plant and of cultivars.

All types of material produced fruits at the third and fourth growing seasons without significant statistical differences among the types of material in terms of yield, on average 0.07 kg nut per tree at the third growing season and 0.3 kg nut per tree at the fourth growing season (**Figure 12**, left top). The T. Francescana cultivar exhibited significantly higher yields compared with the other cultivars, producing approximately 0.12 kg per tree at the third growing season and over 0.5 kg per tree at the fourth growing season (**Figure 12**, left bottom). In the third year, small differences were found in T. Francescana between the types of plant material (**Figure 12**, right top), whereas there were no differences in hazelnut production between the different types of plants within each cultivar in the fourth year (**Figure 12**, right).

**Figure 13:**
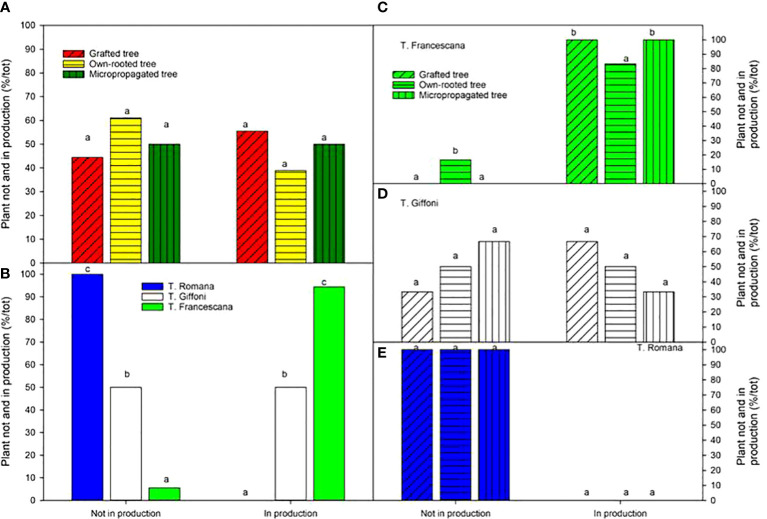
Plant not and in production (bearing > 0.25 kg of nut per tree) at the fourth growing season in different type of plant material (left top) **(A)**, cultivars (left bottom) **(B)**, and interaction (right) **(C–E)**. Different lowercase letters indicate significant differences (P< 0.05) among the average values of type of plant and of cultivars.

When considering plant in production at the fourth growing season, no variations were observed among the different types of plant material, ranging from to 56% for grafted tree to 39% for own-rotted ones (**Figure 13**, left top). Notably, 94% of T. Francescana’s plants were already in production whereas 100% of T. Romana plants and 50% of those of T. Giffoni were not yet in production (**Figure 13**, left bottom).

**Figure 14:**
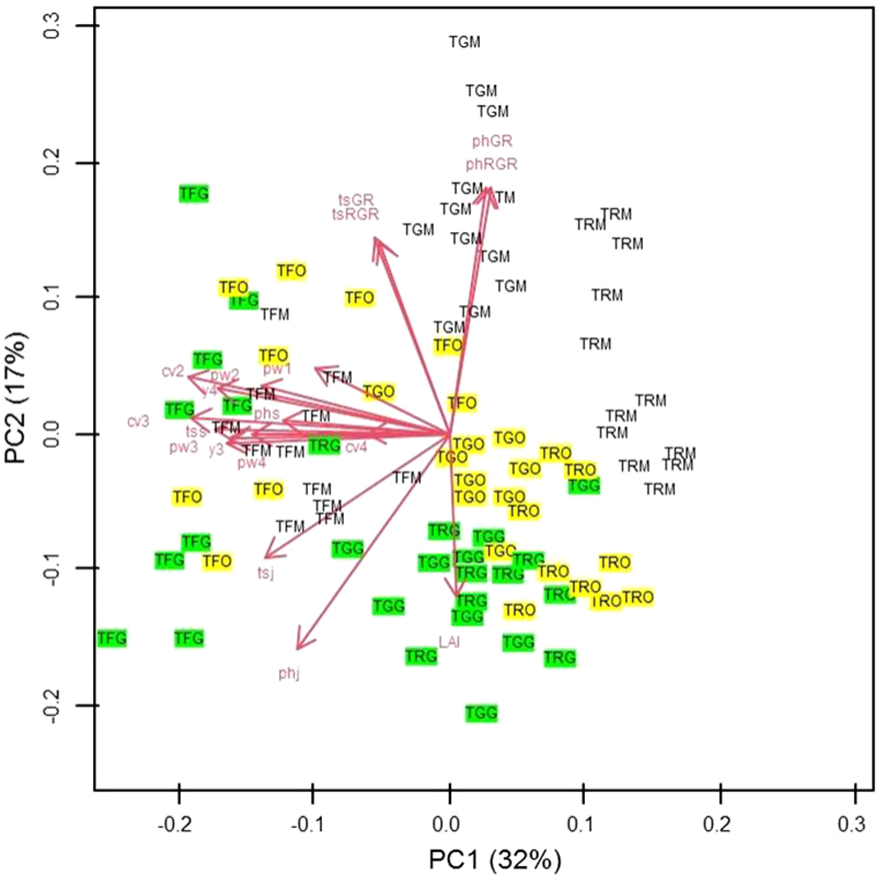
Scatter plot of the scores of Grafted (G) (green coloured), Micropropagated (M) (white coloured) and Own-rooted (O) (yellow coloured) plants of T. Giffoni (TG), T. Francescana (TF) and T. Romana (TR) cultivars on the two-dimensional plane defined by PC1 and PC2 So TGG means grafted plant of T. Giffoni, TGM means micropropagated plant of T. Giffoni and TGO means own-rooted plant of T. Giffoni and so on for the other two cultivars. The main factors analyzed are identified by a code as following: PhGR means Plant Height Growth Rate; PhRGR = Plant height Relative Growth Rate; tsGR = trunk section Growth Rate; tsRGR trunk section Relative Growth Rate; tsj = trunk section January 1^st^ growing season; phj = plant height January first growing season; tss = trunk section September 1^st^ growing season; phs = plant height September 1^st^ growing season; cv2= canopy volume 2^nd^ growing season, cv3 = canopy volume 3^rd^ growing season, cv4 canopy volume 4^th^ growing season, pw1 = pruned wood 1^st^ growing season, pw2 = pruned wood 2^nd^ growing season, pw3= 3^rd^ growing season, pw4= pruned wood 4^th^ growing season, y3 = yield 3^rd^ growing season, yield 4^th^ growing season; LAI = leaf area index 4^th^ growing season.

For PCA results, in the two-dimensional plane defined by the first two principal components (PCs), the primary differentiation among the three propagation types is evident along the diagonal spanning the second and fourth quadrants. Notably, in PC1, which accounted for 32% of the total variance, the significant contributors were the canopy volume during the second and third years, the trunk section measured in September of the first year, and the yield in the fourth year (**Figure 14**). Meanwhile, PC2, explaining 17% of the total variance, exhibited prominent contributions from the growth rate and relative growth rate of plant height and trunk section. Conversely, on the opposite side, plant height measured in January, at the transplantation time, and the leaf area index (LAI) played pivotal roles (**Figure 14**).

Interpreting the PC1–PC2 plot, grafted samples from T. Francescana showed the highest values in aboveground size and yield. In contrast, T. Romana’s micropropagated plants (TRM), positioned to the right on the graph, exhibited diminished size and yield. T. Giffoni’s micropropagated samples (TGM) generally displayed increased values in plant height and trunk section growth rate. T. Giffoni’s grafted (TGG) and T. Romana’s grafted (TRG) plants demonstrated higher LAI values but reduced RGR and GR of plant height and trunk section.

Considerable variability was observed among own-rooted plants of each cultivar. T. Francescana’s own-rooted (TFO) specimens were predominantly distributed on the left side of the plane, whereas T. Giffoni’s own-rooted (TGO) and T. Romana’s own-rooted (TRO) were positioned in the lower-right quadrant (**Figure 14**).

## 4 Discussions

Our research over 4 years has provided valuable insights into the performance dynamics of grafted, micropropagated, and own-rooted hazelnut plants across various Italian cultivars.

Micropropagation is an effective clonal propagation method for many woody plant species and is particularly advantageous for the large-scale multiplication of certified material. This technique addresses issues related to the formation of adventitious species and ensures the genetic fidelity of propagated plants (Bacchetta et al., 2008; Prando Sandoval et al., 2014; Micheli et al., 2019; Leto et al., 2023). Despite its advantages, field cultivation of micropropagated plants sometimes reveals phenotypic differences, likely due to epigenetic changes during the tissue culture processes (Leva et al., 2002). The hesitation to adopt micropropagation commercially for fruit trees, traditionally propagated by grafting or layering, often stems from uncertainties about field performance (Hammershing and Scorza, 1991; Hernandez et al., 1997; Leva et al., 2003; Tetsumura et al., 2004; Leposavić et al., 2016; Chitra et al., 2016; Marín et al., 2023).

Studies across various species have shown mixed results regarding the vigor and uniformity of micropropagated plants compared with grafted ones. For example, micropropagated walnut trees were found to be more vigorous and homogeneous than their grafted counterparts (Cozzolino et al., 2021), and similar observations were made in peach trees (De Souza et al., 2017). A 3-year study on mulberry demonstrated that micropropagated plants and those derived from stem cuttings exhibited no significant quantitative variations, suggesting that both methods can effectively maintain varietal integrity (Chitra et al., 2016). According to the authors, this is because mulberry is a highly heterozygous plant and propagation through axillary buds ensures genetic uniformity and stability among the regenerants.

Jones et al. (1996) observed that micropropagated silver birch trees displayed less variation in height and trunk girth compared with seedling trees. Litwińczuk et al. (2005) reported that in high bush blueberry, softwood cutting plants grew more slowly, produced significantly less, had shorter shoots, and were more variable than micropropagated plants. In peach and Japanese persimmon, micropropagated plants showed either poorer initial growth or smaller increases in trunk diameter compared with budded or grafted trees (Hammershing and Scorza, 1991; Tetsumura and Yukinaga, 1998).

In our study of hazelnuts, micropropagated plants exhibited not only less variability but also greater increases in growth rate compared with both own-rooted and grafted ones in their first growing seasons in the field. This result aligns with the findings of Leva et al. (2002) in olive, where micropropagated plants demonstrated a greater increase in stem leader than the own-rooted plants (cuttings). Additionally, Tetsumura and Yukinaga (1998), in Japanese persimmon, noted that the height of micropropagated plants were initially one-third of the other three types, but by the end of the 3-year experiment, all plants reached the same height. Later, Tetsumura et al. (2004), in a comparative field performance of mature Japanese persimmon grafted vs. micropropagated trees, found that micropropagated trees (M) grew more vigorously than did the grafted trees (G); the differences in tree canopy and trunk cross-sectional area between M and G trees increased annually. This corresponds with the hazelnut data, where micropropagated plants showed higher growth rate than grafted and own-rooted plants, reaching, at the end of the trial, in the fourth growing season, similar canopy volumes to both grafted and own-rooted ones. Salinas et al. (2023), comparing the performance of plantlets of papaya originated by seed, grafting, and micropropagation, found that *in vitro* micropropagated papayas were the least productive, even if they bloomed earlier and set fruit at desirable trunk height, because they were less tall and thick. Hernandez et al. (1997) found in kiwifruit that commercial production first harvest was significantly higher in grafted plants, even if the following years plants obtained from cuttings showed greater vigor and higher cumulative and commercial yield. On the other hand, micropropagated vines showed a later coming into production and significant lower value for all parameters in study (Hernandez et al., 1997). In gooseberry, the plants’ growth vigor and fruit yield were greater in *in vitro*-derived plants than in plants propagated from softwood cutting (Wójcik et al., 2020). In wild cherry (*Prunus avium* L.), in high-bush blueberry (*Vaccinium corymbosum* L.) and in apple (*Malus domestica* Borkh), micropropagated own-rooted cultivars show changes that affect flowering delays or excessive branching (Marín et al., 2023). These effects may be related to the rejuvenation of the plant due to the action of plant growth regulators during tissue culture (Read and Bavougian, 2013), but they were not observed in hazelnut.

In olive, Neri et al. (2020) pointed out how micropropagated trees delay the onset of flowering capacity by only 1 year. Their results showed that the micropropagated trees did not regress to the juvenile stage, but only to a higher level of vigor that ensures a very rapid growth in the post-implant phase with times of onset into production, comparable with those propagated with cuttings (Neri et al., 2020). The differences in production appeared to be related to tree size, particularly in the early years as observed in peach between own-rooted and grafted trees (Hammershing and Scorza, 1991). These results are confirmed in the hazelnut cultivar T. Francescana that, showing a rapid development of the plant, produces nuts from the third year in the field.

In walnut, Hasey et al. (2004) reported a more vigorous growth and higher yield of micropropagated plant of Chandler cultivar to those conventionally grafted onto seedling Paradox rootstock. In fact, own-rooted Chandler trees grew larger than those on Paradox rootstock over eight years and more productive over six years.

Maguylo and Lauri (2004), in apples, pointed out that the reproductive growth was influenced by the combination of root system and genotype. In all cases, grafted trees on M9 rootstock produced more fruit earlier than own-rooted trees but there were differences depending on the genotype. As a general trend, own-rooted trees had a slower entrance into production than trees grafted on M9, with a gap of at least 1 year for first yield.

Contrary to what is observed in a previous trial (Portarena et al., 2022) in the hazelnut, where the grafted plants of T. Francescana, T. Giffoni, and T. Romana cultivars had a slight delay in the entry into production compared with those own-rooted, in this study, no differences in yield have been observed among the three types of plant material. Instead, regarding the three cultivars in trial, the productive results agree with that reported in the literature, that is, the best performances of T. Francescana and T. Giffoni compared with T. Romana especially in the first years of growth (Farinelli et al., 2009).

**Figure 15:**
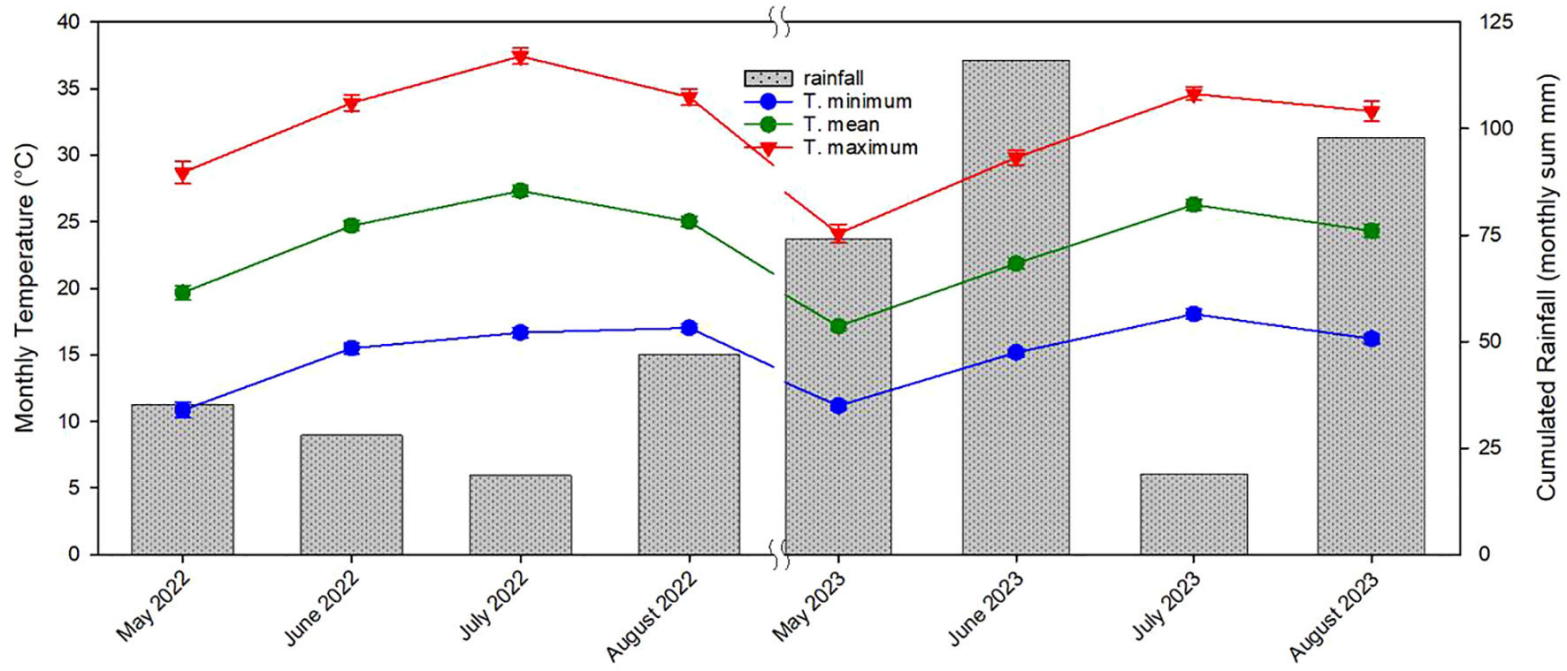
Monthly minimum temperature, monthly mean temperature, monthly maximum temperature and cumulative monthly rainfall recorded from May 2022 to August 2022 and from May 2023 to August 2023 (mean ± s.e.).

The lack of differences in terms of yield observed in this study among the three propagation methods could be attributed to the specific environmental conditions occurred during the third and fourth years of growth. Extended periods of high summer temperatures over the critical threshold and then unusually long period rainy in June and July (approximately 140 mm) decreased the production especially of own-rooted and micropropagated plants (Luciani et al., 2020; Di Lena et al., 2022; Portarena et al., 2022; Farinelli et al., 2023; Portarena et al., 2023; Vinci et al., 2023a) (**Figure 15**). Additionally, the reduced yields observed in the fourth year across all propagation types, as compared with similar earlier studies (Portarena et al., 2023), were likely influenced by these adverse climatic conditions during the critical fruit-setting period (Portarena et al., 2023; von Bennewitz et al., 2019) (**Figure 15**). Tous et al. (1997) observed that over a 4-year study in Spain, own-rooted Negret hazelnut plants demonstrated inferior growth and yield compared with plants grafted onto various rootstocks.

In agreement to Portarena et al. (2022), in this study at the end of the fourth year from plantation, the grafted plants achieved the same size (volume of the canopy) as the own-rooted and micropropagated ones, reaching completely similar values. Although these results were achieved with different growth rates, in fact, in the study of Portarena et al. (2022) during the first 3 years after planting, the grafted plants showed lower vegetative (expressed as volume and pruned wood) growth, whereas, in this study, the grafted plant showed the bigger canopy volume, followed by the micropropagated one, whereas the own-rooted plants were the smaller. These results can be ascribed to the environmental conditions that occurred during the third and fourth years of growth, as reported by Portarena et al. (2023), which pointed out how a deeper root system would provide better access to water resources then appropriately supporting the water demand caused by the intense evapo-transpiration and then allowing the higher assimilation rates of grafted plants in comparison with the own-rooted, especially during summer.

About LAI, the recorded data agree with those of Bignami and Natali (1997), who reported LAI values between 2.39 and 5.21 for a 2-to-3-year-old hazelnut tree of T. Romana cultivar, at the end of the growing season. In comparison, our data were lowest of those achieved in another hazelnut orchard of T. Giffoni and Nocchione cultivar, 6 years old, with a similar plant density of 666 trees/ha, but with a different training system, namely, multisystem bushes (Altieri et al., 2022), instead of single trunk. The observed differences could be attributed to the variations in training system and the younger age of our studied plants, which also exhibited different canopy shapes.

Interestingly, no data are available in literature regarding the LAI of micropropagated hazelnut plants. In our study, we found that the grafted, own-rooted, and micropropagated plants showed different and decreasing LAI values, whereas the canopy volumes were not different. These habits were related to the growth habit; in fact, the grafted plants showed an erect growth habit whereas the own-rooted and micropropagated ones resulted to have a semi-erect canopy. Therefore, the grafted plants had a thicker and higher canopy, whereas the other two types of plants have a wider and lower canopy and therefore a lower LAI.

The PCA confirms the impact of the propagation technique on the expression of studied agronomical traits, revealing distinct responses of each cultivar to the three techniques of propagation, namely, *in vitro* (micropropagated plant), English double cleft winter grafting on *Corylus colurna* L. rootstock (grafted plant), and stump layering (own-rooted plant). The T. Francescana cultivar demonstrates better responses to grafting in terms of both growth and production, maintaining favorable values even in own-rooted and micropropagated propagation methods. Conversely, T. Romana, when obtained through micropropagation, shows lower growth and yield. These results agree with observations in apples, where grafted trees showed earlier and more abundant fruit production compared with own-rooted trees, with variations depending on the genotype (Maguylo and Lauri, 2004).

The PCA further showed that micropropagated plants display greater uniformity compared with grafted ones, whereas own-rooted plants exhibit more variability. This suggests that *in vitro* propagation allows standardizing the plant material, preserving cultivar characteristics (Neri et al., 2020).

The results from our 4-year study support the suitability of *in vitro* propagation for cultivating hazelnut cultivars with high genetic fidelity, a claim that future molecular analyses could substantiate. This finding aligns with observations from other species, where shoot regeneration from apical and axillary buds has shown minimal variability, as seen in gooseberries (Wójcik et al., 2020), date palms (Saker et al., 2006), and olives (Leva et al., 2002). Moreover, the use of *in vitro* propagation promotes the production of healthy and true-to-type materials, improving the economic value of the crop (Bacchetta et al., 2008).

*In vitro* propagation has demonstrated several advantages in hazelnut cultivation. Our research reveals that micropropagated hazelnut plants exhibit uniform growth and do not experience delays in the onset of flowering, performing comparably with grafted and own-rooted plants without exhibiting juvenile effects. This is particularly significant as it suggests that micropropagated hazelnuts can enter productive phases as quickly as those propagated by traditional methods, thereby reducing unproductive periods. The use of certified *in vitro-*derived materials, like those from the new released cultivar T. Francescana^®^, guarantees to transplant high-quality plants. These plants not only show rapid growth but also maintain consistency across generations, which is critical for scaling up production without losing varietal traits.

## Data availability statement

The original contributions presented in the study are included in the article. Further inquiries can be directed to the corresponding authors.

## Author contributions

CT: Data curation, Formal analysis, Investigation, Methodology, Validation, Writing – original draft. SF: Investigation, Writing – review & editing. RB: Data curation, Investigation, Validation, Writing – review & editing. AV: Investigation, Writing – review & editing. SPe: Data curation, Formal analysis, Investigation, Writing – review & editing. MMe: Conceptualization, Methodology, Writing – review & editing. MMi: Conceptualization, Methodology, Validation, Writing – review & editing. FF: Validation, Writing – review & editing. SPo: Data curation, Formal analysis, Validation, Writing – original draft, Writing – review & editing. GD: Conceptualization, Funding acquisition, Methodology, Writing – review & editing. DF: Conceptualization, Funding acquisition, Methodology, Project administration, Supervision, Validation, Writing – original draft, Writing – review & editing.
